# Spatiotemporal Scan and Age-Period-Cohort Analysis of Hepatitis C Virus in Henan, China: 2005–2012

**DOI:** 10.1371/journal.pone.0129746

**Published:** 2015-06-15

**Authors:** Fangfang Chen, Dingyong Sun, Yuming Guo, Wei Guo, Zhengwei Ding, Peilong Li, Jie Li, Lin Ge, Ning Li, Dongmin Li, Zhe Wang, Lu Wang

**Affiliations:** 1 National Center for AIDS/STD Control and Prevention, Chinese Center for Disease Control and Prevention, Beijing, China; 2 Institute for AIDS/STD Control and Prevention, Henan Center for Disease Control and Prevention, Zhengzhou, China; 3 Division of Epidemiology and Biostatistics, School of Public Health, University of Queensland, Brisbane, Australia; University of North Carolina School of Medicine, UNITED STATES

## Abstract

**Background:**

Studies have shown that hepatitis C virus (HCV) infection increased during the past decades in China. However, little evidence is available on when, where, and who were infected with HCV. There are gaps in knowledge on the epidemiological burden and evolution of the HCV epidemic in China.

**Methods:**

Data on HCV cases were collected by the disease surveillance system from 2005 to 2012 to explore the epidemic in Henan province. Spatiotemporal scan statistics and age-period-cohort (APC) model were used to examine the effects of age, period, birth cohort, and spatiotemporal clustering.

**Results:**

177,171 HCV cases were reported in Henan province between 2005 and 2012. APC modelling showed that the HCV reported rates significantly increased in people aged > 50 years. A moderate increase in HCV reported rates was observed for females aged about 25 years. HCV reported rates increased over the study period. Infection rates were greatest among people born between 1960 and 1980. People born around 1970 had the highest relative risk of HCV infection. Women born between 1960 and 1980 had a five-fold increase in HCV infection rates compared to men, for the same birth cohort. Spatiotemporal mapping showed major clustering of cases in northern Henan, which probably evolved much earlier than other areas in the province.

**Conclusions:**

Spatiotemporal mapping and APC methods are useful to help delineate the evolution of the HCV epidemic. Birth cohort should be part of the criteria screening programmes for HCV in order to identify those at highest risk of infection and unaware of their status. As Henan is unique in the transmission route for HCV, these methods should be used in other high burden provinces to help identify subpopulations at risk.

## Introduction

Chronic infection with hepatitis C virus (HCV) is known as the major agent of chronic liver disease, hepatic cirrhosis, and hepatocellular carcinoma. WHO estimated that there are more than 185 million people with chronic liver disease due to HCV infection, and over 350,000 people die from HCV-related diseases worldwide each year [1,2]. HCV is a serious public health problem in many developing and developed countries because of the absence of a prevention vaccine, continued transmission through injecting drug use, unsafe blood and blood products and unsafe medical and non-medical equipment; and lack of curative treatment regimens.

China was a relatively high endemic area of HCV infection in the past [3]. A national survey carried out in 1992 showed that the HCV prevalence was 3.20% in general population in Mainland China, estimated at approximately 40 million HCV infections [4]. According to another multicenter epidemiological study between 1991 and 1995, the average HCV prevalence in general population was over 2.2% [5]. Due to the lack of regulatory and implementation oversight on the safety of blood and blood products, transfusion-transmitted infections (TTIs) including HCV rapidly spread among commercial plasma donors in the 1990’s in central China, effectively establishing the HCV epidemic in the country [6].

Strengthened regulations on blood safety such as mandatory screening of all donated blood for TTIs in 1993 and implementation of voluntary blood donation policies in 1998 reduced the risk of HCV transmitted through blood and blood products [7]. According to a meta-analysis, the pooled prevalence of HCV infection was 12.87% (95% confidence interval, CI: 11.25%– 14.56%) among blood donors before 1998, decreased to 1.71% (95% CI: 1.43%– 1.99%) after 1998 [8].

Several provinces continue to have relatively high HCV prevalence. Since 2003, multiple studies reported increasing HCV cases during the past decades suggesting that the risk of HCV transmission exist [9–13]. There are gaps in knowledge on the epidemiological burden and evolution of the HCV epidemic in China. In this study, we use HCV case-reporting data to explore the spatial and temporal variation of the HCV epidemic in a province with high burden of HCV.

## Materials and Methods

### Source of data

HCV is one of the notifiable infectious diseases according to the Law of Infectious Diseases of China amendment of 2004. A standardized case reporting form (CRF) is used for collection of demographic and diagnostic information, including date of birth, gender, residence, occupation, date of diagnosis etc. All CRFs for newly diagnosed HCV are reported to the China Information System for Disease Control and Prevention (CISDCP) by local physicians and health workers since 2003. CISDCP is a web-based real-time database, which collates case reporting information at the national level by the National Centers of Disease Control (NCDC). All reported HCV cases must be confirmed serologically as antibody-positive for HCV.

Henan is a province in central China and shoulders a large burden of HCV infection, reporting 14.55% (9641/66283) of all reported HCV cases in the country in 2005 and 19.78% (39887/201622) in 2012. The province covers an area about 167 000 km^2^ and 170 counties, and has approximately 94 million population.

Data of all HCV cases reported in this province during January 1^st^, 2005 to December 31^st^, 2012 was extracted from the CISDCP database.

### Data analysis

To measure the intensity of HCV infection in a defined area, we used the index termed as ‘reported rate’; defined as the number of reported cases and resident population as the numerator and denominator, respectively. This index has been used in other articles on HCV case reporting [14,15]. Relative risks (RRs) between particular groups rather than the absolute differences of reported rates were used to depict the spatiotemporal trends of HCV infection.

In order to observe the spatial and temporal characteristics, counts of HCV cases were calculated for each district/county level by calendar year, age, gender, and birth cohort. The district/county of HCV cases were labeled by geocode (latitude and longitude). HCV cases were grouped into eighteen 5-year age groups, from 0 to 85+ years. Demographical data and electronic map were obtained from the information system of the NCDC.

### Age-period-cohort analysis

In order to examine the trend of HCV incidence over time, three factors (age of HCV cases when diagnose, calendar year for HCV diagnosis, and birth year of diagnosed HCV cases) were modelled using Age-Period-Cohort (APC) model [16]. APC model separates the birth cohort and age effect from the calendar time effect [17–19] and has been widely used to assess the trends in disease surveillance [20] such as cancer, diabetes and mortality [21–23].

We used a Poisson regression model to fit the age-period-cohort model. A penalized spline was used for age, and birth cohort. Period and gender were modeled as categorical variables. The model was determined as:

$$\text{log}\left(\mu_{ij}\right)=\alpha +S\left(age_{i},df\right)+\beta period_{j}+S\left(cohort_{k},df\right)+\delta gender_{l}+offset\left(\text{log}\left(n_{ijl}\right)\right).$$

Where S was penalized spline, *df* was degree of freedom. S(age_i_, *df*) (i = 1, …, I) was the age effect, βperiod_j_ was the period effect (j = 1,…J), S(cohort_k_, *df*) was the cohort effect (k = 1,…,K), δgender_l_ was used to control for gender effect, and log(n_ij_) was the offset term for population. We compared seven partial models including null model, age only, period only, cohort only, age + gender, period + gender, cohort + gender. The Akaike information criterion (AIC) was used to decide the model goodness-of-fit and degree of freedom for spline [24,25]. The full model provided the best-fit model with the lowest AIC values (Table 1). The effect of age, period and cohort was expressed as estimated relative risk with two-tailed 95% confidence intervals. All analyses were performed by R software, version 3.1.0 (R Development Core Team, Vienna, Austria). The “mgcv” package was used to perform Poisson regression model.

**Table 1:** Results from the APC modeling.

Model	Variables	AIC	df
0	Null model	445814.8	1.00
1	Age	332515.4	9.98
2	Period	412621.4	8.00
3	Cohort	342639.5	9.96
4	Age + Gender	332478.7	10.98
5	Period + Gender	412361.8	9.00
6	Cohort + Gender	342584.9	10.96
7	Age + Period + Cohort + Gender	299215.8	25.88

### Spatiotemporal scan analysis

The SaTScan software (Version 9.3) was used to perform spatiotemporal scan statistics [26] to detect the temporal and spatial clustering of reported HCV cases. This method identifies the presence, locations and temporal window of the clusters [22] and is widely used to detect clustering in a number of health-related fields [27–30].

The essential principle was to allow circular windows of different sizes to range across the study region. Potential clusters in Henan province was explored by scanning each location (district/county) with a flexible size of the radius of the window from 0 to the upper limit, defined at 50% by default. The window size was determined by the percentage of the total population size in the area. For each location and size of the scanning window, a likelihood ratio was computed for the alternative hypothesis that there was an increased risk of disease inside the circle [31]. The likelihood function was maximized over all window locations and sizes, and the circular windows with the maximum likelihood ratio values were identified as the most likely cluster.

Outputs of this method included a cylindrical window with a circular geographic base with height corresponding time [32]. An associated *P* value, based on Monte Carlo hypothesis testing [33], was used to evaluate whether the cases are randomly distributed. For each simulation, the likelihood ratio statistic was computed and the observed value compared with the set of simulated values to find the significance probability. The number of simulations was restricted to 999. Clusters with statistical significance of *P*<0.05 were reported.

Separate analyses were performed for: pure spatial clusters, spatiotemporal clusters for calendar periods and spatiotemporal clusters for birth cohorts. As the onset of the disease was dependent on age and gender, covariate adjustment in SaTScan was used to identify pure spatial and spatiotemporal clusters. ArcGIS 10.0 (ESRI Inc., Redlands, CA, USA) was used to present the results of clustering in the electronic maps.

### Ethics

This study was approved by the Institutional Review Board of National Center for AIDS/STD Control and Prevention, Chinese Center for Disease Control and Prevention (IRB: 00002276). The analyses used data extracted from the routine CISDCP case reporting database of Henan. Permission for data use was approved by China CDC (Email: data@chinacdc.cn for public use). Information extracted was at population-level and did not include individual patient data or personal identifiers.

## Results

There were 177,171 HCV reported cases in Henan between 2005 and 2012. Only 0.5% (n = 803) of cases were excluded owing to lack of geographic information. The number of HCV reported cases increased by calendar year from 8,123 in 2005 to 39,887 in 2012. The gender ratio of HCV reported cases (male/female) was close to 1, and the median age was 47 years.

### APC study

**Fig 1:**
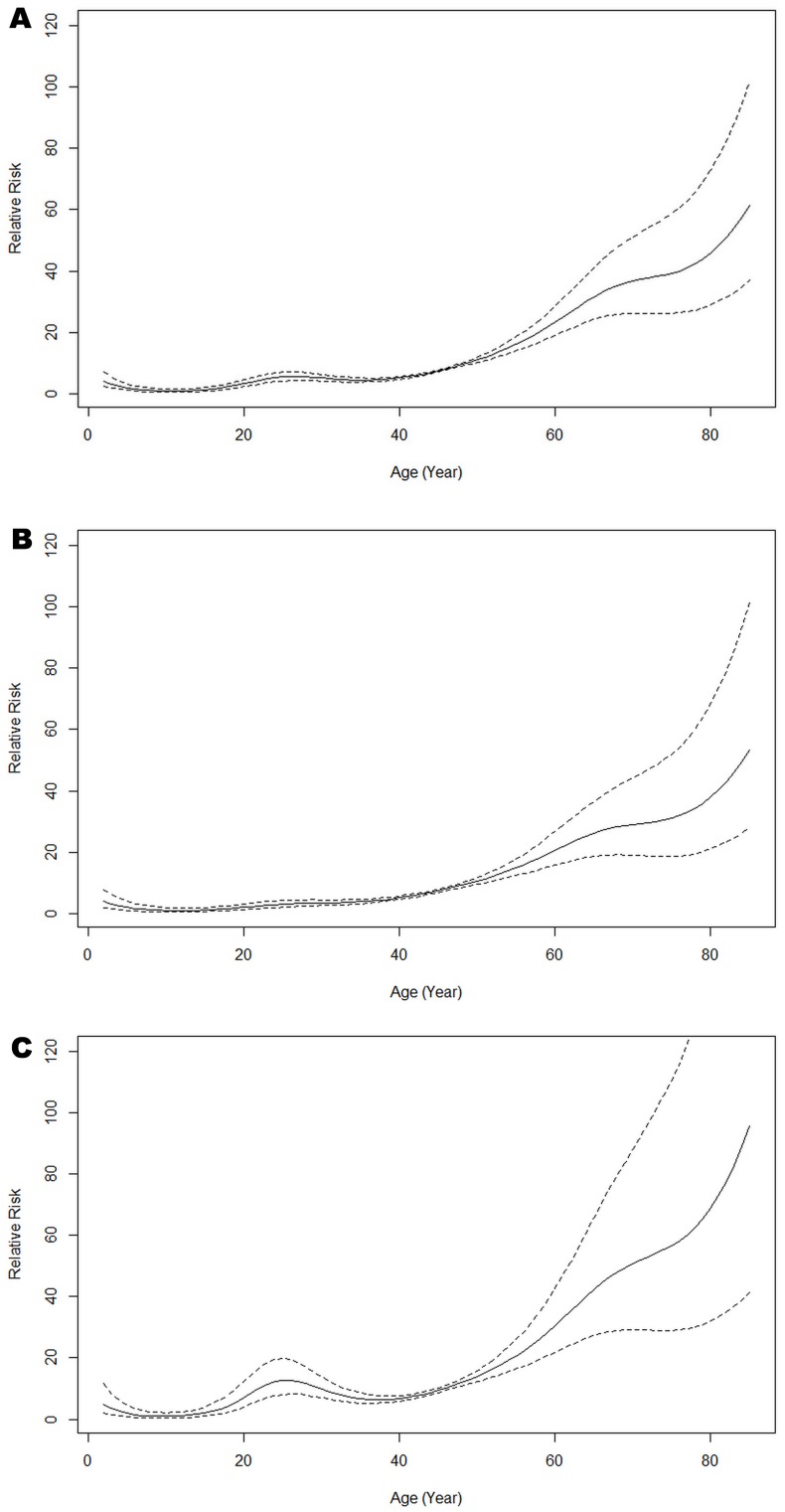
Age effect from the full APC model for all (A), male (B) and female (C). The continuous line = estimated effect of age; dashed lines = 95% confidence interval of the estimates.

**Fig 2:**
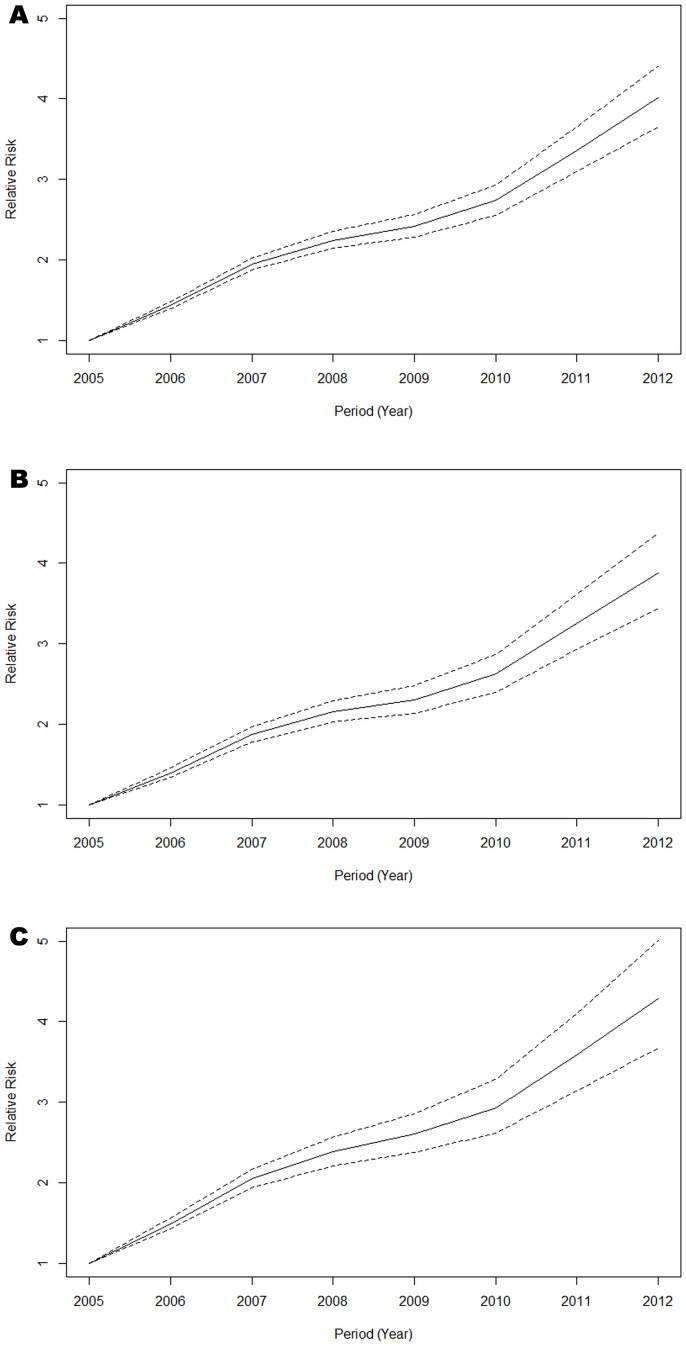
Period effect from the full APC model for all (A), male (B) and female (C). The continuous line = estimated effect of period; dashed lines = 95% confidence interval of the estimates.

Fig 1 shows the age effect on the reported HCV infection rates. Overall reported rates were similar across age-groups until about 50 years of age, thereafter increasing between two to four-fold from 60 to 80 years. Overall, both men and women had similar trends of HCV infection. In women of 25 years of age, however, there was a small but noticeable increase in infection rates. Reported HCV rates increased between 2005 and 2012, with a marked increase from 2010 (Fig 2).

**Fig 3:**
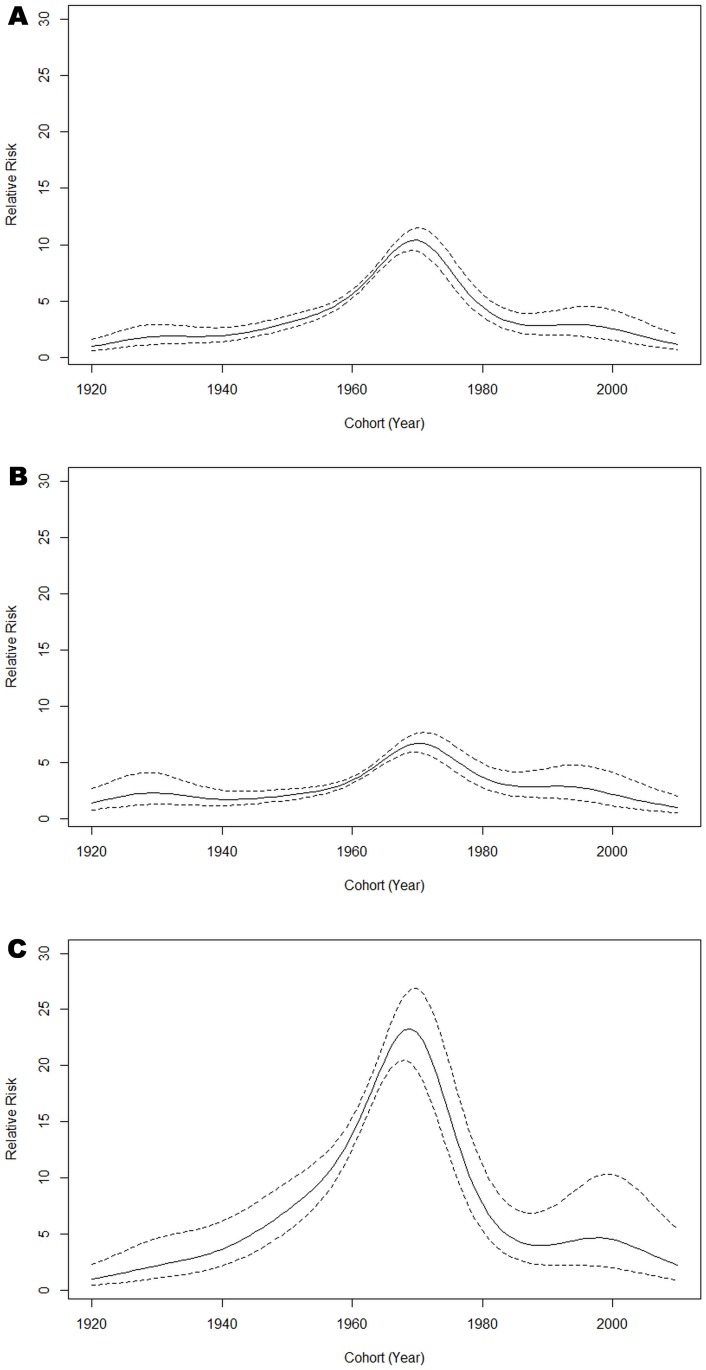
Cohort effect from the full APC model for all (A), male (B) and female (C). The continuous line = estimated effect of cohort; dashed lines = 95% confidence interval of the estimates.

In general, reported HCV rates were higher in birth cohorts born between 1960 and 1980, compared to before 1960 and after 1980 (Fig 3). People born around the 1970s have the greatest rates of infection. Women born between 1960 and 1980 had a five-fold increase in HCV infection rates compared to men, for the same birth cohort. A second smaller peak in HCV infection was seen in women born around the year 2000.

### Spatiotemporal analysis adjusting for age and gender

#### Purely spatial

**Fig 4:**
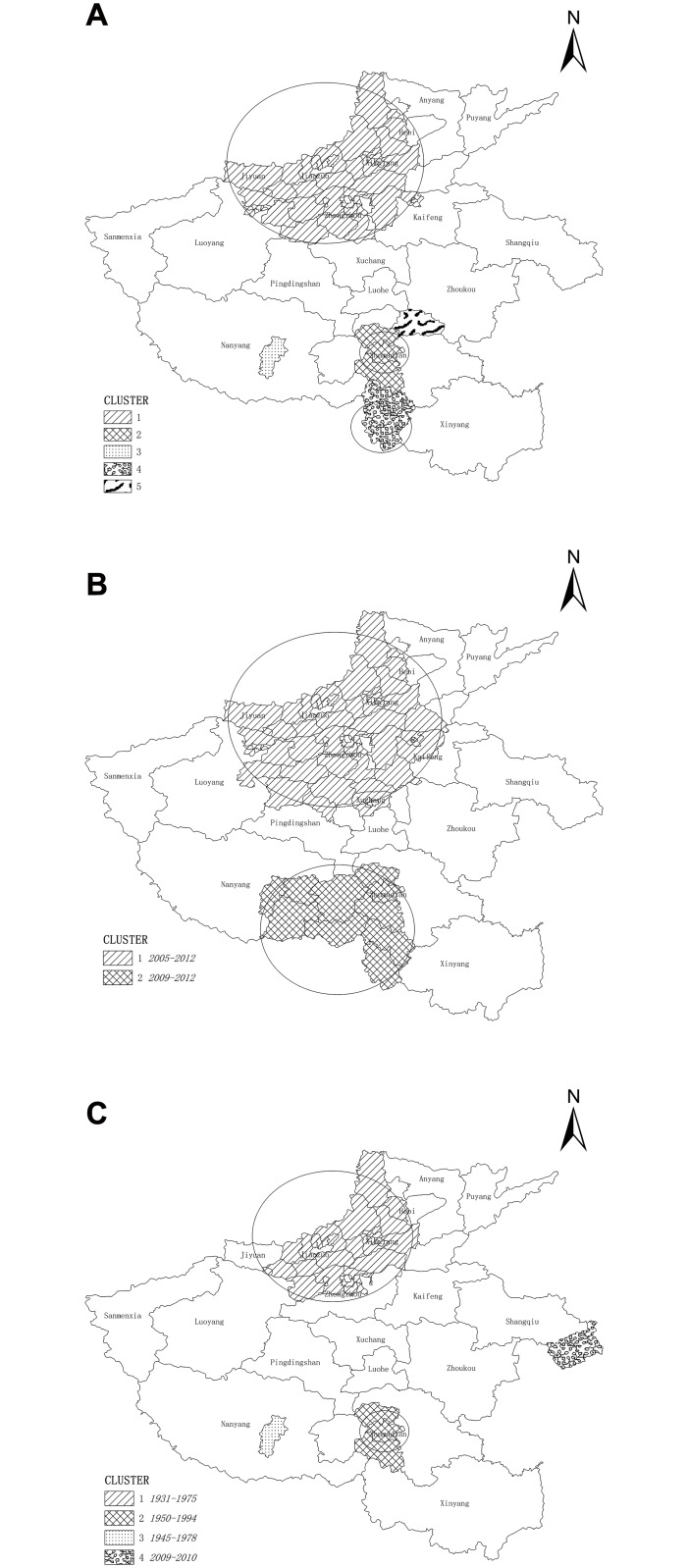
Results from the spatiotemporal scan analysis. (A) Five clusters identified for purely spatial. (B) Two clusters identified for calendar periods and place of living. (C) Four clusters identified for birth cohorts and place of living.

Pure spatial clustering analysis examined five clusters during 2005–2012 (Table 2, Fig 4A). The most likely cluster was centered in 35.2571N, 113.2750E, Shanyang district and encompassing a radius of 108.61 km, including 45 districts/counties. 70103 cases were reported against the 37341.46 expected and the cluster had a relative risk (RR) of 2.46 (P<0.01). Four statistically significant secondary clusters were identified (Table 2).

**Table 2:** Spatial analysis of hepatitis C, using the discrete Poisson model of purely spatial statistic.

Cluster	Cluster center	Radius (km)	No. districts/counties	Population	No. of cases	Expected cases	LLR	RR	P value
1	Shanyang dis	108.61 km	45	19014344	70103	37341.46	15598.70	2.46	<0.01
2	Yicheng dis	27.87 km	3	1505871	7045	3071.02	1921.47	2.35	<0.01
3	Wancheng dis	0 km	1	790250	3708	1637.46	972.50	2.29	<0.01
4	Shihe dis	34.60 km	2	1272970	3808	2548.23	274.50	1.51	<0.01
5	Shangcai	0km	1	1195144	3203	2190.43	207.49	1.47	<0.01

#### Controlling for calendar period

Table 3 and Fig 4B showed the result from the spatiotemporal analysis by calendar period after adjusting for age and gender. Analysis identified two clusters of high infection rates between 2005 and 2012. The first cluster was centered within a 118.02 km radius in the north of Henan province during 2009 to 2012 (RR = 2.60, P<0.01). The other cluster was centered in a radius of 87.75 km in southern Henan during 2010 to 2012 (RR = 2.09, P<0.01).

**Table 3:** Calendar period and spatial analysis of hepatitis C, using the discrete Poisson model of space-time statistic.

Cluster	Cluster year	Cluster center	Radius (km)	No. districts/counties	Population	No. of cases	Expected cases	LLR	RR	P value
1	2009–2012	Wushe	118.02 km	58	26880878	56694	27142.00	15349.08	2.60	<0.01
2	2010–2012	Tongbai	87.75 km	10	6610835	10355	5111.26	2148.25	2.09	<0.01

#### Controlling for birth cohort

Four clusters of elevated risk of HCV infection were found by spatiotemporal analysis for birth cohort (Table 4 and Fig 4C). The first cluster consisted of residents born during the period 1931–1975 in northern Henan (RR = 2.48, P<0.01). Notably, this cluster was similarly located with a size approximated to the cluster found in spatial analysis and controlled for calendar period. The second cluster was found for people born between 1950 and 1994 in the south of Henan (reported cases n = 5,779 against the expected n = 2,237.82; RR = 2.64, P<0.01). The third cluster was found for people born during 1945–1978 in southern Henan. Lastly, the fourth cluster was noted in a specific county in eastern Henan among people born during 2009–2010 in Yongcheng district (RR = 21.39, P<0.01).

**Table 4:** Birth cohort and spatial analysis of hepatitis C, using the discrete Poisson model of space-time statistic.

Cluster	Cluster year	Cluster center	Radius (km)	No. districts/counties	Population	No. of cases	Expected cases	LLR	RR	P value
1	1931–1975	Xiuwu	88.31 km	31	1125921	43125	20369.12	11338.32	2.48	<0.01
2	1950–1994	Yicheng dis.	27.87 km	3	132377	5779	2237.82	1977.77	2.64	<0.01
3	1945–1978	Wancheng dis.	0 km	1	69473	2981	1150.56	1017.10	2.62	<0.01
4	2009–2010	Yongcheng	0 km	1	113015	195	9.13	411.28	21.39	<0.01

## Discussion

Henan has the highest burden of HCV in China [34]. The number of HCV reported cases in this province have gradually increased from 2005 to 2012. To better understand the evolution of the epidemic, we depict the temporal and spatial characteristics of HCV infection from case-reporting data collected during 2005–2012.

HCV case reporting increased gradually per calendar year. This was likely due to scale-up of testing and reporting practices. A slight increase in rates of reporting from 2010 was found and can be attributed to strengthening and special management of HCV surveillance. Since 2009, HCV surveillance was gradually integrated into national HIV/STD surveillance by enhancing case reporting management, adding anti-HCV testing to HIV sentinel surveillance and establishing HCV specific sentinel surveillance sites [35]. In Henan this work has been transferred from the Institute of Immunization Program to the Institute of Prevention and Control of STD/AIDS since 2010, which is responsible for staff training, surveillance and case management, amongst others.

We noted that the HCV infection rates increased with age, especially in older people more than 50 years. This result was similar to previous provincial and national cross-sectional studies in Henan and China [36,37]. This could be a cumulative effect of exposure to HCV transmission risks over a lifetime. Late diagnosis of HCV infection is common because of low awareness and knowledge of HCV such as that found in a national survey [38] and the slow and asymptomatic clinical course of most HCV infections [39]. Our analysis has also detected an apparent birth cohort effect with people born between 1960 and 1980 having the highest risk of infection. This birth cohort may have had specific exposure to HCV. In 1995, commercial plasma donation became popular in poor rural communities in Henan [40] and a previous history of this constitutes a major risk for TTI infection. People born in 1960 to 1980 were in their young adulthood or working during this time and may have been exposed to HCV through this route.

Interestingly, we noted gender differences of HCV infection risk. Women born between 1960 and 1980 had a five-fold increase in risk compared to men. In men, this risk of HCV infection was only slightly higher for those born between 1960 and 1980, compared to those born before or after these years, respectively. These variations among men and women suggest differences in risks and risk behavior. In the traditional culture of central China, men and women had different roles in family and society. Men were considered responsible for their family and were usually the main breadwinner. Driven by economic necessity, commercial plasma donation could have been a financial source. Women after their reproductive age or having finished their main childcare responsibilities may be driven by these same interests. Younger women may have been exposed to HCV infection through unsafe blood or blood products during pregnancy, labour and delivery; or unsafe medical practices during this time.

Spatiotemporal analysis results showed a large cluster of HCV cased in northern Henan for which the birth period was 1931–1975. Secondary clusters were located around Zhumadian in southern Henan, where most cases were born in 1950–1994. The lack of overlap in the birth cohorts suggests that the HCV epidemic in the north evolved much earlier than in the south.

Northern Henan has a long history of a strong and developed economy with established medical facilities compared to other cities in the province, and is the major hub for in-migration from other areas [41]. Higher and earlier reporting of HCV may be due to better access of health care, testing and better clinical capacities for diagnosis. High demand of blood may have fueled commercial plasma donation earlier in this area.

In southern Henan, HCV infection occurred in a mainly rural population with an undeveloped economy, mostly from former plasma donation [42]. A survey of one village with more paid plasma donors in this region found that, HCV prevalence in paid plasma donors and other villagers were 73.76% and 8.04%, higher than that of general population in China (3.20%), and their prevalence has also increased with the total times of paid plasma donation [43].

Lastly, a specific county confined to Yongcheng in 2009–2010 was found in the secondary clusters by birth year and space analysis. People born during this specific interval period and living in this region had very high infection risk. During this period, an outbreak of HCV infection occurred in Guoyang county of Anhui province and Yongcheng city of Henan province in late November, 2011 [44]. This local event was related to use of intravenous infusion and unsafe medical injection, two of which were the most common treatments in primary care in China [45]. WHO reported that unsafe injection is the most common cause of HCV injection in developing and transitional countries, causing two million new infections each year and accounting for 42% of cases [46]. A study of 1000 HCV cases registered in a hospital found that 8% of HCV infection were acquired from previous nosocomial infection such as hemodialysis, operation, injection, intravenous infusions for therapy and dental treatment [47]. Similarly, nosocomial infection of hepatitis C has been reported in other countries such as Egypt [48], France [49], Vietnam [50], Canada [51].

In summary, the age-period-cohort model and spatiotemporal scan methods applied to case reporting data and is useful in understanding the epidemiology and evolution of HCV epidemic. Our study confirmed differences of HCV risk for birth cohorts, men and women and clustering of cases in northern and southern Henan. In order to improve screening and detect those at highest risk, birth cohort should added as a criteria for testing in the national programme. It is critical that blood safety be strengthened as well as safe medical injection practices in health settings.

### Strengths and limitations

To the best of our knowledge, this is the first study which uses spatiotemporal scan and the APC model to analyze HCV case reporting data. These methods help to elucidate the distribution and trends of HCV infection in spatial and temporal dimensions. The analysis of HCV infection over time and across geographic regions has an important role in disease surveillance and implications for programme approaches.

We used ‘reported rate’ as the measurement of disease occurrence and especially focus on trends of relative risks (RRs) of HCV infection between groups. We took into account the background population in detecting spatiotemporal distribution of HCV and standardize HCV reported cases to the underlying population at risk, which was helpful to better understand potential factors influencing the occurrence in this region. Determining the true incidence of HCV infection (i.e., the rate of newly acquired infections) was difficult because most acute infections are asymptomatic, and available diagnostic assays do not distinguish acute from chronic or resolved infection [3].

The data used in this study relies on routine disease surveillance which has its limitations. Case reporting is influenced by program and health system factors including the extent of prevention efforts and access to diagnostic facilities. There are known issues with case reporting data such as reporting biases, duplicate and underreporting. Duplicate reporting of HCV reported cases in Henan in a previous survey was shown to be about 4–5% each year [52]. Moreover, data was extracted only for eight years from 2005–2012 which may be relatively limited in order to show more marked temporal trends.

We are unable to use this analysis to draw a clear etiology conclusion using case reporting data. However, the approach used was useful to explore possible reasons for HCV cases as is reported in a geographical region since there is inadequate information on the epidemic. Understanding possible routes or transmission factors help to optimize prevention and control efforts, and better target geographical areas with higher burden of HCV.

### Additional research questions

As the route of transmission in Henan is unique to the central provinces of China, further studies should be conducted to understand the epidemiology and evolution of HCV in other provinces with substantial burden of infection.
