# Correction: Spatiotemporal Scan and Age-Period-Cohort Analysis of Hepatitis C Virus in Henan, China: 2005-2012

**DOI:** 10.1371/journal.pone.0136333

**Published:** 2015-08-19

**Authors:** Fangfang Chen, Dingyong Sun, Yuming Guo, Wei Guo, Zhengwei Ding, Peilong Li, Jie Li, Lin Ge, Ning Li, Dongmin Li, Lu Wang, Zhe Wang

The eleventh and twelfth authors are listed out of order. The correct order is: Fangfang Chen^1^, Dingyong Sun^2^, Yuming Guo^3^, Wei Guo^1^, Zhengwei Ding^1^, Peilong Li^1^, Jie Li^2^, Lin Ge^1^, Ning Li^2^, Dongmin Li^1^, Lu Wang^1^ and Zhe Wang^2^

^1^ National Center for AIDS/STD Control and Prevention, Chinese Center for Disease Control and Prevention, Beijing, China

^2^ Institute for AIDS/STD Control and Prevention, Henan Center for Disease Control and Prevention, Zhengzhou, China

^3^ Division of Epidemiology and Biostatistics, School of Public Health, University of Queensland, Brisbane, Australia
