# Correction: Correction: Spatiotemporal Scan and Age-Period-Cohort Analysis of Hepatitis C Virus in Henan, China: 2005-2012

**DOI:** 10.1371/journal.pone.0139180

**Published:** 2015-09-23

**Authors:** The PLOS ONE Staff

There is an error in the correction published on August 19, 2015. The author order on the originally published article is correct.
